# Regulated Expression of *PTPRJ*/CD148 and an Antisense Long Noncoding RNA in Macrophages by Proinflammatory Stimuli

**DOI:** 10.1371/journal.pone.0068306

**Published:** 2013-06-28

**Authors:** Richa K. Dave, Marcel E. Dinger, Megan Andrew, Marjan Askarian-Amiri, David A. Hume, Stuart Kellie

**Affiliations:** 1 The University of Queensland, Institute for Molecular Bioscience, Brisbane, Australia; 2 The University of Queensland, Cooperative Research Centre for Chronic Inflammatory Diseases (CRC-CID), Brisbane, Australia; 3 The University of Queensland, Australian Infectious Diseases Research Centre, School of Chemistry and Molecular Biosciences, Australia; 4 The University of Queensland Diamantina Institute, Brisbane, Australia; 5 Garvan Institute of Medical Research, Darlinghurst, Australia; 6 The Roslin Institute, University of Edinburgh, Roslin, Scotland, United Kingdom; St. Jude Children’s Research Hospital, United States of America

## Abstract

PTPRJ/CD148 is a tyrosine phosphatase that has tumour suppressor-like activity. Quantitative PCR of various cells and tissues revealed that it is preferentially expressed in macrophage-enriched tissues. Within lymphoid tissues immunohistochemistry revealed that PTPRJ/CD148 co-localised with F4/80, indicating that macrophages most strongly express the protein. Macrophages express the highest basal level of *ptprj,* and this is elevated further by treatment with LPS and other Toll-like receptor ligands. In contrast, CSF-1 treatment reduced basal and stimulated *Ptprj* expression in human and mouse cells, and interferon also repressed *Ptprj* expression. We identified a 1006 nucleotide long noncoding RNA species, *Ptprj-as1* that is transcribed antisense to *Ptprj*. *Ptprj-as1* was highly expressed in macrophage-enriched tissue and was transiently induced by Toll-like receptor ligands with a similar time course to *Ptprj*. Finally, putative transcription factor binding sites in the promoter region of *Ptprj* were identified.

## Introduction

Macrophages are key cells that recognise, ingest and destroy foreign microorganisms and their products as part of the innate immune system. Macrophages have an additional role as antigen-presenting cells, and so are central to the optimal functioning of both the innate and acquired immune response [1]. Pathogen-associated molecular patterns (PAMPs) such as bacterial endotoxin (LPS) and CpG DNA and other toll-like receptor (TLR) ligands, induce the release of proinflammatory products such as cytokines and chemokines, thereby enhancing pathogen clearance [2]. Ligation of surface receptors commonly activates protein phosphorylation cascades, which are mediated by the selective activation of protein tyrosine kinases (PTKs). Such responses are usually transient and can be negatively regulated by protein tyrosine phosphatases (PTPs) [3]–[5], and perturbation of the balance between PTK and PTP activity may result in a failure of inflammation to resolve or dysregulation of cell proliferation, which can lead to life-threatening chronic inflammatory diseases or malignancy [6]–[11]. An example of this is the constitutive tyrosine phosphorylation of the PI3 kinase/Akt pathway due to the reduction in the activity of SHP-1 in the allelic moth-eaten viable (Me*^v^*/Me*^v^*) mouse, resulting in severe autoimmunity [12].

PTPRJ (CD148, DEP-1, PTPη, Byp or PTPβ-like tyrosine phosphatase) is a type-III receptor PTP containing a single cytoplasmic phosphatase domain and an extracellular domain containing eight to twelve FNIII repeats, depending on species [13]. PTPRJ is found in a wide range of cell types [14], [15] and evidence for a tumour suppressor role has been indicated due to its reduced expression in some malignant tumours, its regulation by cell density, and the reversion of the transformed phenotype when PTPRJ function is restored [16]–[21]. PTPRJ has several substrates, including PDGF β-receptor [22], hepatocyte growth factor (HGF) receptor [23], vascular endothelial growth factor (VEGF) receptor-2 [24] and the p85 subunit of PI3-kinase [25].

Within human and mouse tissues, macrophages exhibit the highest expression of *Ptprj* [26]–[30] ^,^ and in this cell type *Ptprj* expression is up-regulated by LPS and down-regulated by CSF-1 [30]. Knockout of *Ptprj* indicates a positive role in monocyte activation as it dephosphorylates the negative regulatory tyrosine in *src* family kinases in a manner similar to CD45 [31], [32]. Little is known about the molecular mechanism underlying the regulation of *Ptprj* expression. Although there is little data on regulation of transcription, the 5′ end of the mRNA is thought to attenuate translation [33].

Recently, we characterized differential expression of *Ptprj* in normal and cancerous human breast tissue and in the developing mouse mammary gland [21]. Microarray analysis of the *Ptprj* gene locus identified seven probes that targeted long noncoding RNA species originating from the first intron [21], [34]. Although previously regarded as “junk”, it is now becoming increasingly understood that ncRNAs play a vital role in regulating and co-ordinating the developmental complexity of eukaryotic organisms [35]. The differential and developmental specificity of long ncRNA (lncRNA) expression, in combination with the widespread conservation of their promoters, splice sites and primary sequence [36], suggests that they are generally functional [37]–[39]. The molecular mechanisms of lncRNA functions are diverse and cannot be easily generalised, and unlike protein-coding genes, their function cannot currently be predicted from their primary sequence [40]. Previous functional studies of lncRNAs reveal that they can act by influencing target gene expression at specific genomic loci, either by directly interacting with chromatin regulatory proteins or by modulating the activity of their interacting partners [41]–[43]. While lncRNAs play important roles during normal cellular development and differentiation [44], lncRNAs are also associated with several diseases, including heart disease, Alzheimer’s disease, psoriasis, and cancer [45]. In addition to our observation of multiple lncRNAs originating from the *Ptprj* locus, the potential for RNA regulation of *Ptprj* was also recently highlighted by a report showing that *PTPRJ* is negatively regulated by the short RNA microRNA328 [46].

Here we investigate the expression of *Ptprj* and an antisense lncRNA originating from the *Ptprj* locus in macrophages. We show that *Ptprj* is highly expressed in macrophage-enriched tissues, that it is upregulated in response to various toll-like receptor ligands and downregulated by CSF-1. We further show that the expression of *Ptprj-as-1*, an lncRNA that is transcribed antisense to the coding sequence, is regulated by proinflammatory factors in a manner similar to the *PTPRJ* gene. Finally we characterise the promoter region of human and mouse *Ptprj* and identify putative transcription factor binding sites. An understanding of the biology of phosphatases such as *Ptprj* in macrophage-specific signalling cascades may enable the identification of key endogenous regulators of inflammation and therapeutic targets for inflammatory diseases.

## Materials and Methods

### Ethics Statement

All animal handing and treatment protocols were approved by the University of Queensland Animal Ethics Committee, certificate 570-08. Mice were monitored daily for any adverse reactions and euthanized by exposure to CO_2_.

### Animal Handling

Bone marrow-derived macrophages (BMMs) were derived from cells obtained from the femurs of carbon dioxide euthanized C57BL/6 mice in accordance with University of Queensland ethics guidelines. Thioglycollate-elicited peritoneal macrophages (TEPMs) were obtained by injecting 6–8 week-old male mice with 1 mL of 10% w/v thioglycollate broth and recovering the peritoneal exudate by peritoneal cavity lavage with 10 mL of phosphate-buffered saline (PBS) 5 days post injection as described previously [47].

### Cell Culture

RPMI 1640 medium (Invitrogen Life Technologies) supplemented with 10% FCS (JRH Biosciences), 20 U/mL penicillin (Invitrogen Life Technologies), 20 µg/mL streptomycin (Invitrogen Life Technologies), and 2 mM L-glutamine (Invitrogen Life Technologies) (complete medium) was used for culture of RAW 264.7 cells and bone marrow-derived macrophages (BMMs). Briefly, bone marrow cells were cultured for 7 days in complete medium in the presence of 10,000 U/mL of recombinant human CSF-1 (Chiron Corporation, Emeryville, CA, USA) on bacteriological plastic plates. RAW 264.7 [48] cells were maintained on bacteriological plates (Sterilin, Staffordshire, UK) in complete media containing 5% heat-inactivated fetal bovine serum for a maximum of four weeks in culture. RAW 264.7 cells were passaged with an 18-gauge hypodermic needle and syringe. NIH3T3 and HEK 293 cell lines were maintained in D-MEM (Dulbecco/Vogt Modified Eagle’s Minimal Essential Medium, GIBCO) supplemented with 2 mM L-glutamine (Glutamax-1, Invitrogen), 10% heat-inactivated fetal bovine serum (JRH Biosciences, Lenexa, KS, USA), 20 U/mL penicillin and 20 µg/mL streptomycin (Invitrogen). The cells were cultured in T75 filter cap flasks (Nunc). All primary cells were maintained in a 37°C incubator containing 5% CO_2_. Lipopolysaccharide (from Salmonella Minnesota, Sigma Aldrich, St. Louis, MO, USA) was used at a final concentration of 10 ng/mL. Recombinant human colony stimulating factor-1 (a gift from Chiron, Emeryville, CA, USA) was used at a final concentration of 10^4^ U/mL. Phorbol 12-myristate 13-acetate (PMA) (Sigma Chemical Co.) was used at a final concentration of 10^−7 ^M. Where shown, cells were treated with 10 µg/mL of anti-CD148 antibody or hamster isotype control antibody (Serotec).

### Quantitative Real Time PCR

RNA extraction from mammalian cells or tissues was performed using the RNeasy mini kit (Qiagen, Valencia, CA, USA) as per the manufacturer’s instructions. RNA was quantified by spectrophotometry at 260 and 280 wavelengths. RNA purity was ensured by an A_260_/A_280_ of at least 1.8. Genomic DNA contamination was removed from RNA preparations using DNase (Ambion, Austin, TX, USA) and cDNA was synthesised from 2–5 µg of total RNA using Superscript III (Invitrogen, Carlsbad, CA, USA), using oligo-dT primers (Geneworks, Adelaide, Australia). cDNA was quantitated using SYBR Green (Applied Biosystems, Foster City, CA, USA) in 20 µL reactions in a 96 well plate. Each cDNA was quantified in experimental triplicate. No amplification controls (a minus-reverse transcriptase control and a minus sample control) were included in each reaction plate to ensure the absence of contaminating genomic DNA. Data was collected and analysed using the ABI Prism software. Gene expression was determined relative to *Hprt* (hypoxanthine-guanine phosphoribosyl transferase) mRNA using the comparative threshold method as previously described [21]. Calculations were performed in Microsoft Excel following equations provided by Applied Biosystems. Unless otherwise stated, error bars in figures indicate the standard deviation of duplicate cDNA quantitation in the same thermal cycle run. Expression profiles were typically quantitated in at least two separate experiments (as stated in the figure legends) using completely independent preparations of cells and RNA extracts. Primers (f, forward; r, reverse) used were as follows: mouse *csf1r* f: CCAGAGCCCCCACAGATAA, r: AGCTTGCTGTCTCCACGTTTG; *human csf1r f:* CCTTCAGGAGCAGGCCCAAG, *r:* CCTTGCTCGCAGCAGGTCAG; *mouse Ptprj* f: CAGTACAGTGAATGGGAGCACTGAC, r: GTCCGTATTCTGCCACTCCAACT; *human Ptprj* f: AGTACACACGGCCCAGCAAT, r: GAGGCGTCATCAAAGTTCTGC; *mouse Ptprj-as1* f: CCATCTCCCATT GTCCAAAC, r: TGATTGAAGGACAGCTGGAA *mouse Hprt* f: GCAGTACAGCCCCAAAATGG, r: AACAAAGTCTGGCCTGTATCCAA; *human Hprt f:* TCAGGCAGTATAATCCAAAGATGGT, r: AGTCTGGCTTATATCCAACACTTCG.

### Antibodies

Monoclonal hamster anti-mouse CD148 antibody was generated as described [14] and affinity purified with Protein G sepharose. This antibody was a gift from Prof. Arthur Weiss (Howard Hughes Medical Institute, Rosalind Russell Medical Research Centre for Arthritis, University of California-San Francisco, San Francisco). Rat anti-mouse Mac-2 monoclonal antibody (un-purified culture supernatant from clone TIB-166 hybridoma, ATCC) was kindly provided by Dr Andrew Cook, University of Melbourne. Antibodies to the following proteins were purchased from commercial sources: F4/80 monoclonal antibody from Serotec, goat anti-hamster IgG biotin conjugated (as a secondary against hamster anti-mouse CD148 antibody) from Pierce and goat anti-rat IgG biotin conjugated (as a secondary against rat anti-mouse F4/80 and Mac-2 antibodies) from Serotec.

### Immunoblotting

Total protein extracts were prepared using the SDS-boiling method [26]. Cells were washed twice with ice cold PBS and lysed on the culture plate with 500 µL boiling lysis buffer (66 mM Tris-HCl pH 7.4, 2% SDS) per 1×10^6^ cells. Extracts were homogenised by repeated passage through a 26-gauge needle and further boiled for 5 minutes. Protein concentration of total lysates was determined using the BCA assay kit (Pierce, Rockford, IL, USA). Extracts were resolved by SDS-PAGE (4–12%), transferred to Imobilon-P (Millipore, North Ryde, NSW, Australia), blocked and probed with anti-CD148 antibody or antiphosphoprotein antibodies in the presence of phosphatase inhibitors. Blots were washed, probed with HRP-labelled secondary antibodies (Cell Signaling Technology) or Streptavidin-HRP-labelled (Pierce) tertiary antibody in case of CD148 blots, and detected using enhanced chemiluminescence (ECL) reagents (Amersham Pharmacia Biotech, Piscataway, NJ, USA). Membranes were stripped with 66 mM Tris-Cl (pH 6.7)/2% SDS/100 mM 2-mercapto-ethanol and reprobed with total Akt antibodies (Cell Signaling Technology).

### Immunohistochemistry

Immunohistochemistry was performed using an immunoperoxidase technique with diaminobenzidine (DAB) as the chromogen as described previously [21], [49]. Expression of CD148, F4/80 and Mac-2 were examined in serial sections. Briefly, sections were deparaffinized and rehydrated followed by antigen retrieval. For CD148, microwave antigen retrieval was performed in citrate buffer pH 6.0 for 2 min and allowed to cool overnight. For F4/80 Carezyme Trypsin digestion kit (Biocare Medica) was used as per the manufacturer’s instructions. Sections were washed in Tris buffered saline (TBS) and endogenous peroxidase activity was blocked by incubating the sections in 3% H_2_O_2_ (diluted in TBS) for 30 min. Sections were incubated for 60 min in serum block [10% fetal calf serum (FCS) plus 10% normal serum (species of secondary antibody) in TBS] and then treated with the primary antibody for 60 min. Sections were subsequently incubated for 30 min with a biotinylated F(ab′)2 fragment of goat anti-rat or rabbit anti-hamster immunoglobulin (DakoCytomation), followed by horseradish peroxidase (HRP)-conjugated streptavidin (DakoCytomation) and developed with DAB chromogen (DakoCytomation) according to the manufacturer’s specifications. The specificity of the staining was confirmed by using matched isotype control antibodies. All sections were counterstained with Mayer’s haematoxylin. Slides were allowed to dry on the bench before mounting using permanent mounting media (Cytoseal, Stephens Scientific). All incubations were carried out at 25°C sections were washed between each step with TBS. Slides were examined and photographed using a transmitted light microscope (Olympus BX-51, with DP-70 camera).

### Statistical Analysis

One-way analysis of variance (ANOVA) was used to determine any significance differences between the samples. In addition significance differences between individual time points for each group were calculated using a two-tailed independent Student’s t-test. Data sets yielding a p value greater than 0.05 were regarded as not statistically different.

## Results

### *Ptprj* is Preferentially Expressed in Macrophage-enriched Tissues and Cell Types

**Figure 1:**
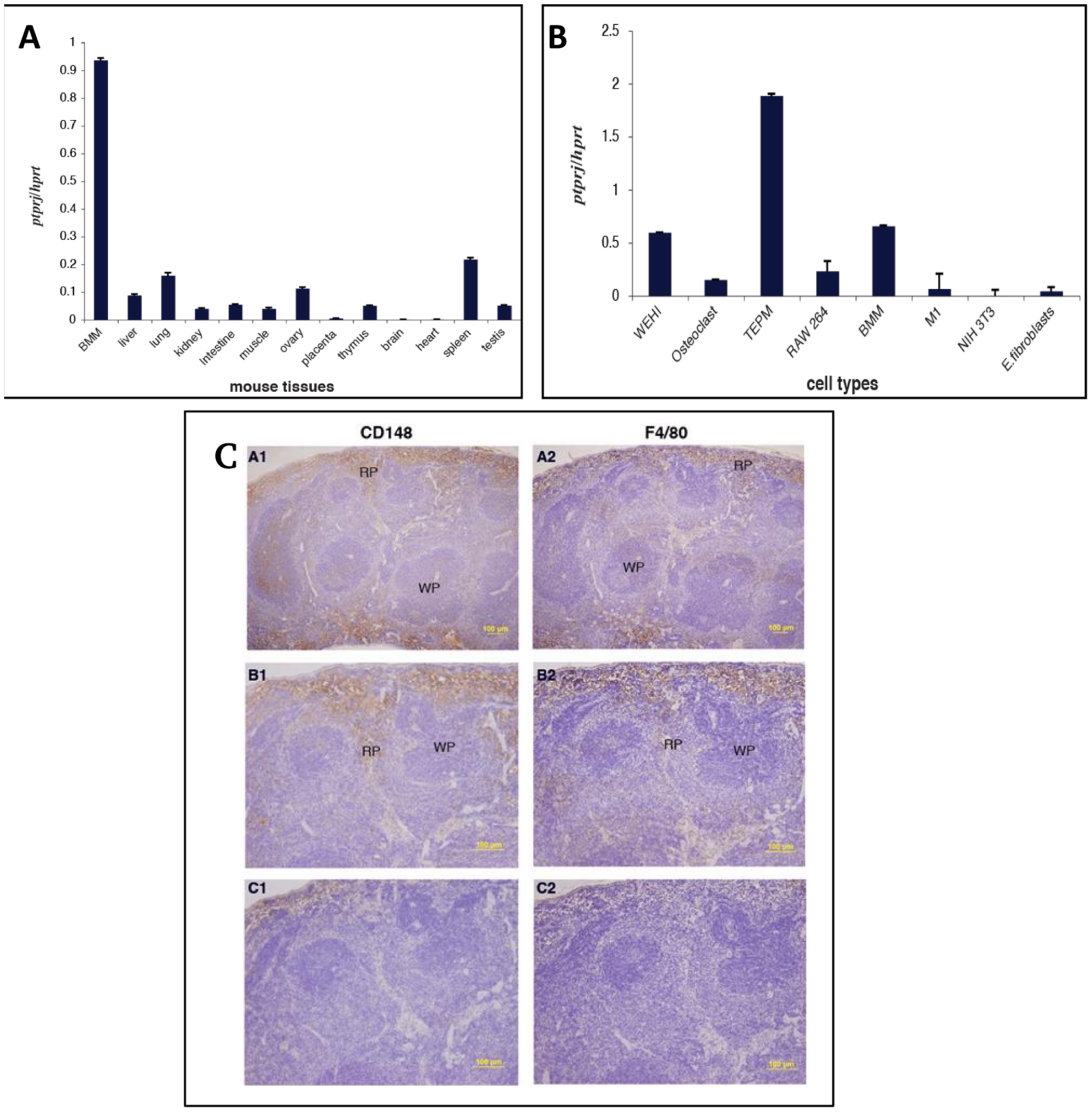
Ptprj mRNA expression in murine tissues. (A). qPCR of *Ptprj* from RNA extracted from mouse tissues. (B) qPCR of *Ptprj* from RNA from pre-B lymphoid cell line WEHI-231, osteoclast-like cell line (RAW 264.7, C4), TEPM, macrophage-like cell line (RAW 264.7), BMM, myeloid cell line M1 and fibroblasts (NIH3T3 and mouse embryonic). (C). Immunohistochemistry of cell-specific expression of PTPRJ in mouse spleen sections. Sections were immunostained for CD148 (A1, B1) or F4/80 (A2, B2) and with CD148 (C1) and F4/80 (C2) isotype control antibodies. All sections were counterstained with haematoxylin. RP, red pulp; WP, white pulp. Original magnification: x100 (A), x200 (B, C). Bar, 100µm.

To characterize the expression of *Ptprj* in various mouse tissues, quantitative real-time PCR analysis was performed. *Ptprj* expression was highest in bone marrow derived macrophages (Figure 1A). Tissue distribution revealed that *Ptprj* was high in tissues with a significant macrophage content. In particular, expression of *Ptprj* was highest in the spleen. However, other macrophage-rich tissues such as lung, liver, kidney, intestine, thymus, ovary, muscle and testis showed detectable expression levels of *Ptprj*. Minimal expression of *Ptprj* was detected in brain, heart and placenta. The expression of *Ptprj* in a range of different cell types was also examined. *Ptprj* mRNA was expressed at relatively elevated levels in inflammatory (TEPMs) and primary macrophages (BMMs) and at lower levels in the macrophage-like cell line RAW 264.7, differentiated RAW/C4 osteoclast-like cells and myeloid precursor cell line M1 (Figure 1B). *Ptprj* mRNA levels were low in pre-B lymphoid cell line WEHI-231, embryonic fibroblasts and *Ptprj* mRNA was virtually undetectable in the fibroblast cell line NIH 3T3.

As *Ptprj* expression was highest in spleen compared to other tissues (Figure 1A), immunohistochemistry was performed to identify the cell types within spleen that express the protein product of *Ptprj*, CD148. CD148 expression pattern in the spleen was restricted to the red pulp region, and was indistinguishable from the expression pattern of F4/80, a mononuclear phagocyte marker (Figure 1C). This confirmed the macrophage lineage–specific expression of CD148 in the mouse spleen and was consistent with the quantitative real-time PCR data (Figure 1A,B).

### Regulation of Ptprj mRNA and Protein in Response to Inflammatory Stimuli in Mouse Macrophages

**Figure 2:**
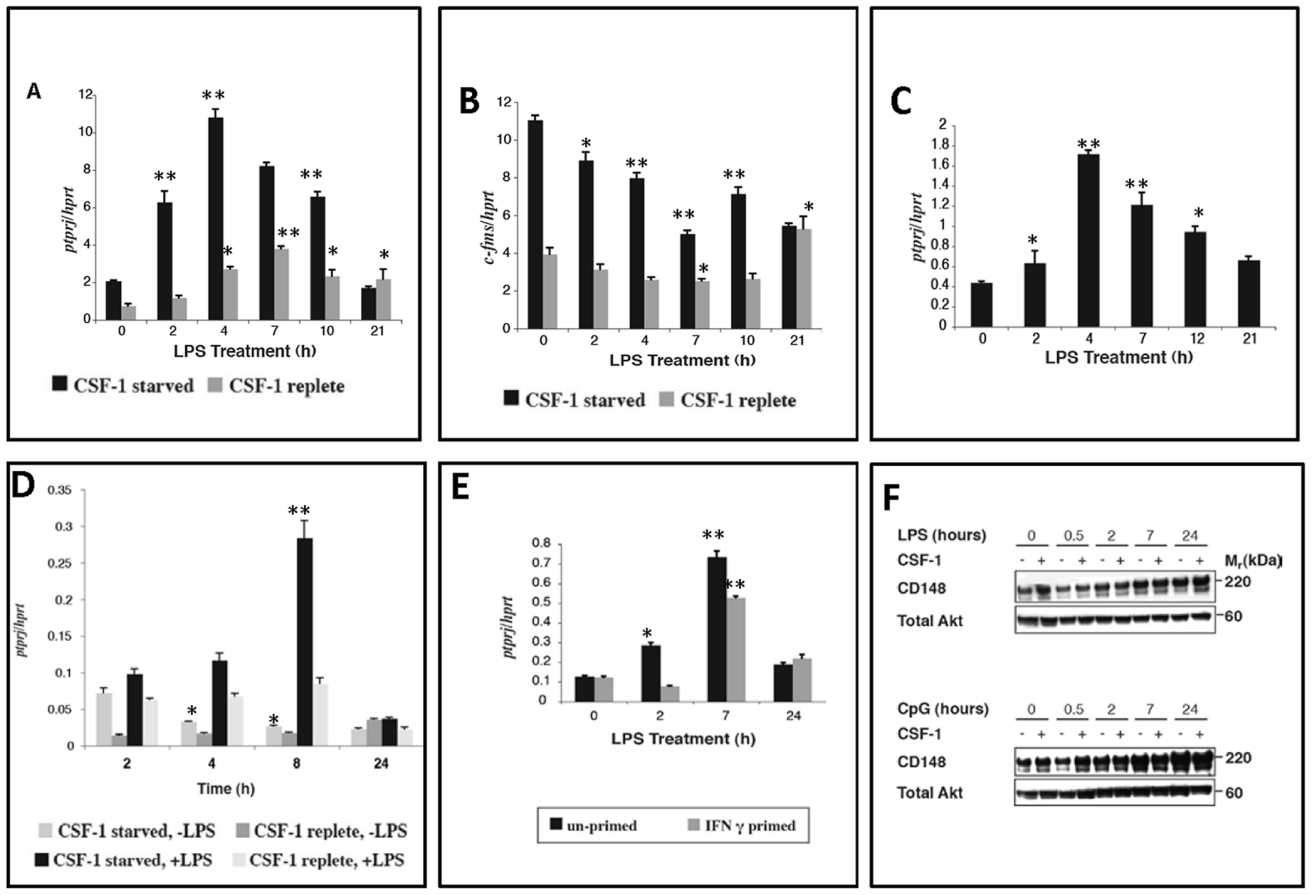
Regulation of *Ptprj* expression in mouse macrophages by proinflammatory stimuli. A–C: Regulation of *Ptprj* expression by CSF-1 and LPS in mouse bone marrow-derived macrophages (BMMs). BMMs were maintained overnight in the presence or absence of CSF-1 (1×10^4^ U/mL) before treatment with LPS (10 ng/mL) (A, B). RAW 264.7 cells were maintained overnight in the absence of CSF-1 before treatment with LPS (10 ng/mL) (C). *Ptprj* (A, C) and *c-fms* (B) expression profiles were assessed by quantitative real-time PCR. Profiles are representative of two independent experiments. D: Regulation of *Ptprj* expression by CSF-1 and LPS in mouse thioglycollate-elicited peritoneal macrophages (TEPMs). TEPMs were maintained overnight in the presence or absence of CSF-1 (1×10^4^ U/mL) before treatment with LPS (10 ng/mL). *Ptprj* expression profile was assessed by quantitative real-time PCR. E: IFNγ treatment of bone marrow derived macrophages suppresses the LPS mediated induction of *ptprj*. BMMs were maintained overnight in the presence of CSF-1 (1×10^4^ U/mL) and presence or absence of IFNγ (500 pg/mL) before treatment with LPS (10 ng/mL). RNA was extracted at each time point and used for the synthesis of cDNA. *Ptprj* expression profile was assessed by quantitative real-time PCR. Datapoints (+/− SD) represent the average of triplicate samples each from triplicate independent experiments. Significance values were determined by one-way analysis of variance (ANOVA). *denotes p<0.05; **denotes p<0.005; n = 3. F: Regulation of *PTPRJ* protein in response to LPS, CpG DNA and CSF-1. BMMs were maintained overnight in the presence or absence of CSF-1 (1×10^4^ U/mL) before treatment with LPS (10 ng/mL) [top panel] or CpG DNA (0.1 µM) [bottom panel]. Protein lysates were separated by SDS-PAGE, transferred to PVDF membranes and immunoblotted for PTPRJ. The membrane was then stripped, and reprobed for total Akt as a loading control. Profiles are representative of two independent experiments.

**Figure 3:**
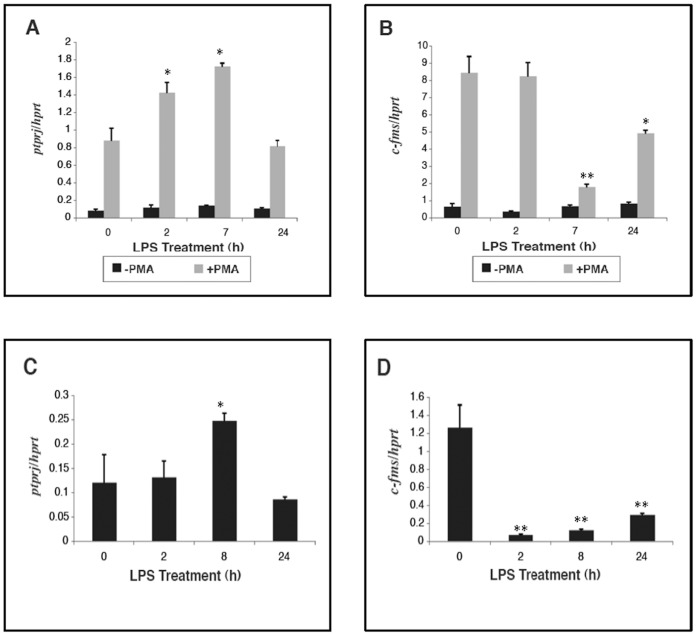
*Ptprj* expression in response to LPS in human mononuclear phagocytic cells. THP-1 cells were maintained for 24 hours in the presence or absence of PMA (10^−7^ M) to induce differentiation, before treatment with LPS (10 ng/mL) (A, B). Human dendritic cells were treated with LPS (10 ng/mL) over a time course (C, D). *Ptprj* (A, C) and *c-fms* (B, D) expression profiles were assessed by quantitative real-time PCR. Datapoints (+/− SD) represent the average of triplicate samples. Significance values were determined by one-way analysis of variance (ANOVA). *denotes p<0.05; **denotes p<0.005; n = 3. *denotes p<0.05; **denotes p<0.005; n = 3.

Quantitative real-time PCR analysis of BMMs stimulated with LPS showed regulated expression of *Ptprj*. In the absence of CSF-1, LPS induced *Ptprj* mRNA expression approximately six-fold peaking at 4 hours. However, the presence of CSF-1 caused a marked reduction in both the basal and LPS-induced expression levels of the *Ptprj* transcript (Figure 2A). As expected, the expression pattern of *c-fms*, a classical marker for mature, differentiated macrophages was suppressed by CSF-1 (Figure 2B). To investigate other murine macrophage populations, the expression profile of *Ptprj* was examined in the macrophage-like RAW 264.7 cell line and in thioglycollate-elicited peritoneal macrophages (TEPM). Although, the pattern of *Ptprj* induction in response to LPS in RAW 264.7 cells was identical to that of BMMs (Figure 2C), the fold induction was less compared to BMMs. Because RAW 264.7 cells are deficient in cell surface expression of CSF-1R [50], *Ptprj* mRNA expression in these cells was examined only in the absence of CSF-1. As with BMMs, in TEPM *Ptprj* was strongly induced by LPS and this was repressed by CSF-1 (Figure 2D). Pre-treatment of macrophages with IFNγ (priming) results in a more rapid and heightened response to LPS and other TLR agonists [48], [51]. Unlike LPS and CpG DNA, IFNγ alone had no effect on induction of *Ptprj* in BMMs (data not shown). Conversely, priming of BMMs with IFNγ repressed LPS-mediated induction of *Ptprj* mRNA expression at early time points (Figure 3E).

To determine whether *Ptprj* was regulated at the protein level, western blot analyses of BMM cell lysates from LPS and CpG time courses were performed (Figure 2F). CSF-1 increased the basal level of CD148 protein (0 h lanes), however CD148 protein levels increased with time after treatment with LPS in both replete and CSF-1-starved cells. Although CpG DNA showed a similar basal pattern of induction of CD148 by CSF-1, an increased level of CD148 protein with time after stimulation was observed. Thus both qPCR (Figure 2D) and western blotting (Figure 2F) showed an increase in CD148 mRNA and protein respectively, albeit with a different timecourse.

### Regulation of *PTPRJ* Gene Expression in Human Mononuclear Phagocytic Cells

The expression profile of *PTPRJ* in response to LPS was also examined in the human monocytic cell line THP-1 and in human dendritic cells. Unlike mature differentiated macrophages such as RAW 264.7 cells or BMMs, THP-1 cells are premonocytes that are committed to the monocytic lineage, but are non-adherent and lack many macrophage-specific cell surface markers. Therefore, for the induction of terminal differentiation to macrophage-like cells, THP-1 cells were cultured in the presence of PMA. PMA mimics some actions of CSF-1, but activates distinct signalling pathways [52]. In the absence of PMA, LPS stimulation had no effect on *PTPRJ* expression (Figure 3A). However, presence of PMA led to a dramatic induction of *PTPRJ* in response to LPS. Even in the absence of LPS, PMA induced basal *PTPRJ* expression approximately ten-fold, whereas in the presence of LPS, the induction was even stronger. PMA strongly induced *c-fms*, consistent with the view that PMA imparts a macrophage-like phenotype to these monocytic cells, however LPS treatment suppressed c-fms expression in PMA-differentiated cells (Figure 3B).

CD148 has been recognised as an accessory molecule present on the surface of peripheral blood dendritic cells [53]. Although overall fold induction was lower than in mouse macrophages, LPS induced *PTPRJ* expression in human peripheral blood dendritic cells (PBDCs) (Figure 3C). As with PMA-differentiated THP_ cells, *c-fms* was down-regulated by LPS in human dendritic cells (Figure 3D).

### Expression of Long Noncoding RNA *Ptprj-as1* during Macrophage Activation

#### Expression of *Ptprj-as1* in murine tissues

**Figure 4:**
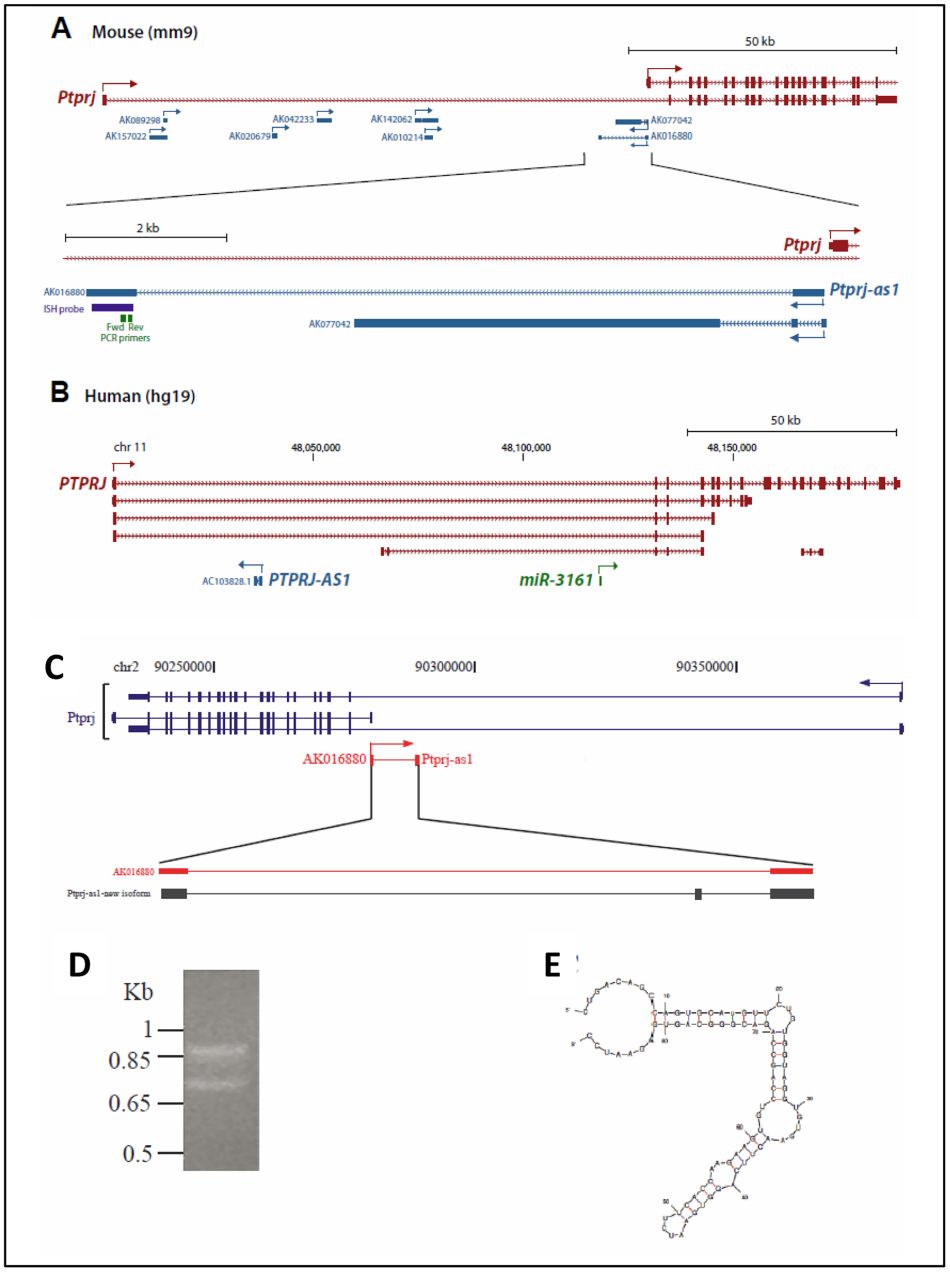
Characterisation of *Ptprj-as1.* A–B: *Ptprj-as1* maps to the reverse strand within the boundaries of the murine *Ptprj* gene. Comparison of the mouse Ptprj (A) and human PTPRJ (B) loci. Protein coding transcript isoforms of Ptprj/PTPRJ are shown in red and long noncoding transcripts are shown in blue. Arrows indicate the direction of transcription. The human microRNA miR-3161 is shown in green. Position of PCR primers used for qRT-PCR for mouse Ptprj-as1 are indicated. C-D: Mapping (C) and expression (D) of a splice variant of murine *Ptprj-as1* in brain, kidney and testis. E: Predicted secondary structure of *Ptprj-as1* splice variant.

In a previous study, we examined microarray expression profiling data of mammary epithelial cells derived from pregnant, lactating and involuting mice [34]. The microarray that this dataset is based upon features probes targeting ∼29,550 mRNAs and 8,693 long ncRNAs. Within this dataset, we identified seven probes that targeted long ncRNAs that originated from the *Ptprj* locus [21]. All of these long ncRNAs occurred within the first intron of *Ptprj*; two were on the antisense strand and the remaining five were on the sense strand (Figure 4A). On the basis of its high expression in monocytic cells, cytoplasmic localization, and spliced character, we selected the lncRNA that we term *Ptprj-as1* (GenBank Accession ID AK016880) for further examination. As the other lncRNAs were expressed at low levels or unexpressed in monocytic cells, and they were derived from unspliced transcripts, they were not pursued for further characterization as such low-expressed single-exon transcripts are challenging to study because they cannot be easily distinguished from genomic DNA in expression studies.

*Ptprj-as1* is a spliced 1,006 nt lncRNA that is transcribed antisense to *Ptprj-as1* and is expressed at levels similar to *Ptprj*. Interestingly, *Ptprj-as1* is antisense to the 5′UTR of a short isoform of *Ptprj* that lacks the canonical first exon, raising the possibility that *Ptprj-as1* may be co-expressed with the short isoform of *Ptprj-as1*. *Ptprj-as1* was identified as a long noncoding RNA of unknown function that is transcribed from the reverse strand of the *Ptprj* gene (Figure 4A). Note that the *PTPRJ-AS1* in human is in a different location to *Ptprj-as1* in mouse and should not be considered as an orthologue (Figure 4B). RT-PCR analysis of the murine transcript in different tissues confirmed the existence of tissue-specific splice variants (Figure 4C). Spleen, brain, and testis revealed two isoforms of *Ptprjas-1*, one; as reported previously [21] and a second that contains an additional 80 base exon (Figure 4D). The examination of the secondary structure of the additional exon showed a stable stem loop structure (Figure 4E).

#### Expression of *Ptprj-as1* in murine macrophages

**Figure 5:**
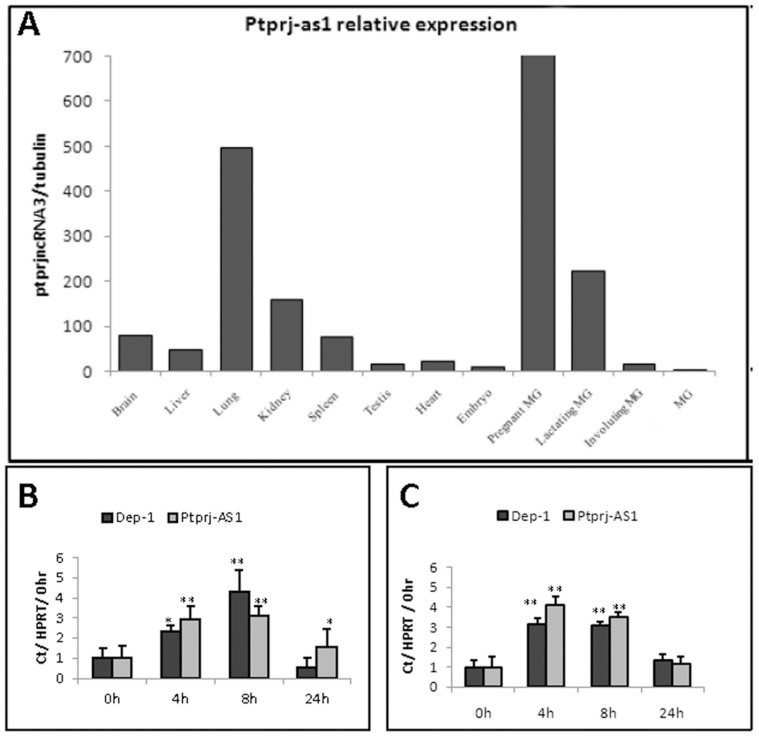
Expression of *Ptprj-as1* in murine tissues and in response to TRL ligands. A: Expression of *Ptprj-as1* in murine tissues. B, C: *Ptprj* and *Ptprj-as1* mRNA expression in BMMs in response to LPS (B) or Pam3Cys (C). mRNA expression was quantified by qRT-PCR and expressed as fold change compared with untreated (0h). Plots represent mean fold change +/− SD; n = 3. Significance values were determined by one-way analysis of variance (ANOVA). *denotes p<0.05; **denotes p<0.005; n = 3. *denotes p<0.05; **denotes p<0.005; n = 3.

*Ptprj-as1* was readily detectable by qRT-PCR (Figure 5A). Preliminary screens of murine tissues demonstrated that whilst *Ptprj-as1* was expressed in many tissues, the transcript showed comparatively elevated levels in lung, brain, kidney and spleen; all tissues that have a sizeable macrophage component (Figure 5A). In unstimulated cells, LPS activation of murine bone marrow-derived macrophages (BMMs) induced a transient increase of *Ptprj* mRNA, peaking at a 4 fold increase 8 hours post addition of LPS. In parallel with *Ptprj*, *Ptprj-as1* expression was also transiently induced by LPS activation of BMMs (Figure 5B). Furthermore, Pam3Cys induced a very similar expression pattern of *Ptprj* and *Ptprj-as1* (Figure 5C). The comparable expression trends of *Ptprj* and *Ptprj-as1* during mBMM activation is consistent with the developing hypothesis that the *Ptprj* and *Ptprj-as1* are regulated by the activity of the same promoter.

### Bioinformatic Identification of Regulatory Sequences in the *Ptprj* Gene

To further investigate the potential mechanisms that direct the expression of *Ptprj*, bioinformatic analysis to identify putative promoter regions was performed.

#### Identification of Ptprj putative promoter by CAGE analysis

CAGE (cap analysis of gene expression) analysis identified the dominant start sites for transcription of the mouse and human Ptprj genes, which in turn allowed functional alignment of the promoters. Ptprj appears to have only one promoter, and both the mouse and human Ptprj promoters lack a TATA box and are rich in GC (data not shown). Whilst the majority of the macrophage-specific promoters also lack a TATA box [54]–[56] the Ptprj promoter differs from typical macrophage promoters such as c-fms [57], [58] in that these generally lack GC-rich elements. Analysis of the Ptprj-as1 promoter did not reveal the presence of either a TATA-box or GC-rich element, suggesting that it may be regulated independently to Ptprj.

### Identification of Putative Transcription Factor Binding Sites

**Figure 6:**
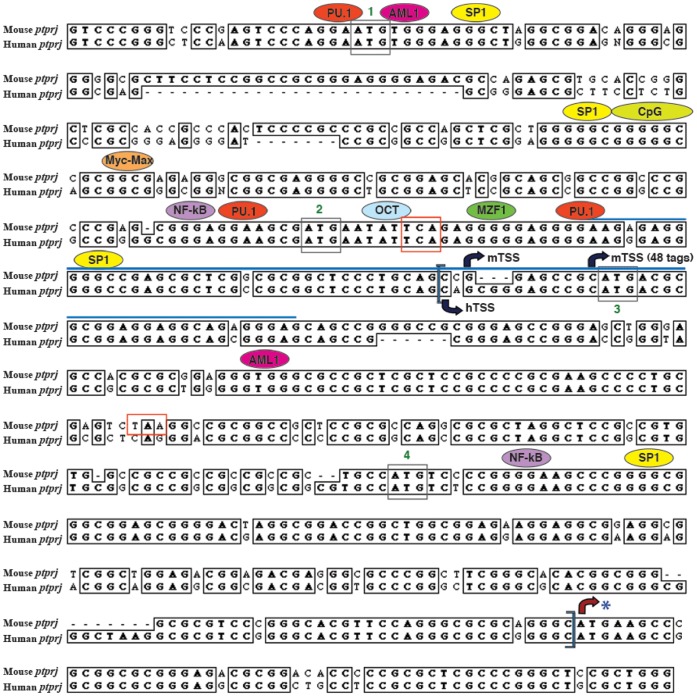
Clustal W alignment of the mouse and human *Ptprj* putative promoters. Consensus transcription factor binding sites conserved in sequence and position between mouse and human were predicted using RVista browser and TRANSFAC. Only the myeloid-specific transcription factors have been shown in this figure. Evolutionary conserved region (ECR1) lies within the blue brackets. Blue line marks the region of transcription start site (TSS) cluster represented in Figure 6. mTSS refers to the transcription start site for mouse *ptprj* and hTSS to the transcription start site for human *Ptprj* predicted by CAGE analysis. The four uAUG codons are highlighted within grey boxes and the stop codons within red boxes. Asterisk (*) represents the translation start site. The boxes represent the conserved sequences between mouse and human. N represents any nucleotide.

The mouse and human sequences corresponding to ECR1 were aligned using ClustalW alignment. This sequence was further examined for consensus transcription factor binding sites conserved in sequence and position between mice and humans using the RVista browser and TRANSFAC (Figure 6). This analysis revealed the presence of multiple transcription factor binding sites that are conserved in both sequence and position between the mouse and human putative promoters. These include many transcription factor binding sites that are commonly functional in myeloid promoters including PU.1, SP1, myeloid zinc finger protein (MZF1), nuclear factor kappa B (NF-κB) or p50, octamer factor binding sequences (OCT) and acute myelogenous leukemia (AML1). The presence of a CpG island upstream of the transcription start site might permit epigenetic control of transcriptional activity of the *Ptprj* promoter [58], [59]. One important observation of the assignment of TSS by CAGE is that the *Ptprj* mRNA is longer than previously appreciated. As a consequence, there may be up to four potential initiation codons or AUGs (uAUGs) in the 5′ untranslated region (UTR) upstream of the putative start site of translation of CD148.

## Discussion

*PTPRJ* has been widely accepted as an epithelial cell expressed gene and has been proposed to be a tumor suppressor in this cell type. However, we show here that *Ptprj* is highly expressed in macrophages and its expression is regulated in response to CSF-1 and proinflammatory stimuli. *Ptprj* expression was highest in spleen, lung, ovary, liver, kidney, intestine, thymus and testis; tissues that are rich in resident macrophage population. Indeed, approximately 18% of the cells in the spleen (the highest expressing tissue for *Ptprj*) are macrophages [60]. Others have shown CD148 is expressed in macrophages and related tissues of human origin [61]. The macrophage-enriched expression of CD148 was further confirmed by the strong correlation of the expression pattern of CD148 and F4/80 surface markers on the splenic macrophages in the red pulp. qRT_PCR showed a transient increase in CD148 mRNA after stimulation with proinflammatory factors such as LPS, and that both basal and stimulated expression is suppressed by CSF-1. Western blotting showed a general sustained increase in CD148 protein after LPS stimulation but no detectable suppression by CSF-1. Although the reason for this discrepancy is unknown, lack of correlation between RNA and protein may be related to the half-life of the protein within the cell. For example, if CD148 has a long half-life (as many membrane/cytoskeletal proteins do), then a short-term down-regulation in mRNA levels would not immediately translate into a down-regulation of protein. On the contrary, protein with a long half-life would continue to appear up-regulated due to the accumulation of the protein in the cell before degradation.

Whilst there have been a number of studies investigating the role of *Ptprj* in both epithelial and haematopoietic cells, its exact role in cell function has not been defined. Overall, *Ptprj* appears to be a negative regulator of cell proliferation in epithelial cells and fibroblasts, by down-regulating receptor tyrosine kinase activity [16], [17], [20]. In contrast, *Ptprj* plays a positive role in the activation of B cells and monocytes by dephosphorylating the negative regulatory carboxyterminal tyrosine of *src* family kinases (SFK), thereby activating these enzymes and potentiating a signalling cascade. *Ptprj* expression has been reported to be up-regulated in epithelial cells as they reach confluence and is down-regulated in some tumours [16], [17]. Although loss of heterogeity has been reported for colon, lung, breast and thyroid cancers [62], we have not detected any major down-regulation of PTPRJ protein or mRNA in a large cohort of breast cancers [21].

Clearly, the regulation of *Ptprj* is of profound importance in a number of cell types, however the regulation of PTPs in general and *Ptprj* in particular is poorly understood [11]. *Ptprj* translation has been shown to be regulated by the use of an alternative start site at the 5′ end of the gene [33]. There is growing evidence that microRNAs (miRNAs) can target the expression of both oncogenes and tumour suppressor genes and recently miRNA-328 has been reported to decrease *Ptprj* expression in epithelial cells [46]. In contrast to the relatively few miRNAs that have been identified, it appears that there are at least as many long noncoding RNAs (lncRNAs) as coding RNAs in the human genome [45], [63], and it is likely that many of these can regulate gene expression [64]. We have identified a lncRNA, *Ptprj-as1*, that is transcribed off the *Ptprj* gene locus and is co-regulated with *Ptprj* coding transcripts in murine monocytes. Although we have not investigated any direct roles of *Ptprj-as1* in regulating *Ptprj*, its tissue-specific expression and co-incident location with *Ptrpj* in the genome, raise the possibility that it may have some role in the expression or splicing of *Ptprj*. Previous studies have revealed that antisense long ncRNAs can directly affect the expression or alternate splicing of nearby genes. For example, expression of a long ncRNAs antisense to the tumor-suppressor gene *p15* results in silencing of the *p15* gene by inducing heterochromatin formation [65]. In another example, differential expression of the lncRNA *LUST*, which originates antisense to an intronic region of *RBM5*, regulates the expression of *RBM5* splice variants [66]. It also noteworthy that *Ptpre*, a related tyrosine phosphatase-encoding gene, also features a contextually analogous antisense noncoding RNA, *Ptpreas* [67]. Finally, although examination of the corresponding Ptprj loci in the human genome revealed a number of lncRNAs, because the human PTPRJ loci differs considerably in terms of both splice variants, exon number, primary sequence, and promoter initiation, it is not possible to identify orthologous lncRNAs. This in itself is not surprising, as lncRNAs seldom reveal identifiable primary sequence conservation, and as an intrinsic feature of their regulatory function as RNAs are thought generally to show great plasticity in their sequence constraints compared to protein-coding genes [37].

CAGE analyses revealed the presence of clustered transcription start sites in both mouse and human *Ptprj* promoters, including conserved multiple upstream AUG (uAUG) codons in the 5′ UTR (data not shown). These uAUGs are common features of mRNAs that encode regulatory proteins. These upstream open reading frames (uORFs) encode for upstream peptides (uPEPs), which are highly evolutionarily conserved and are important for regulating translation of the main CDS [68], [69]. It is conceivable that one of these AUGs is the preferred start codon, instead of the one (marked with asterisk in Figure 6) recognised by the publicly available sequence databases. As shown in Figure 6, there is a conserved stop codon in the 5′ UTR and another in the mouse putative promoter. In addition, translational analysis initiating from the fourth uAUG encodes for a 52 amino acid long peptide in case of mouse *Ptprj*.

As there was a similar pattern of *Ptprj* gene regulation across a range of mouse and human macrophages, a combination of bioinformatic data mining and functional analysis was used to dissect the potential mechanisms underlying the transcriptional regulation of *Ptprj*. NF-κB binding sites were identified in the *Ptprj* promoter (Figure 6). NF-κB has a key role in inflammation as it regulates apoptosis, cell-cycle progression, proliferation and cell differentiation [70], [71]. The responsiveness of both mouse and human *Ptprj* genes to LPS and CpG DNA could therefore be attributed to the presence of an NF-κB binding site in the *Ptprj* putative promoter.

The *Ptprj* promoter is a GC-rich TATA-less promoter, which is the major promoter class in mammals and is a more broad and evolvable category of promoters compared to the TATA box-containing promoters [59]. A number of multiple transcription factor binding sites that are conserved in both sequence and position between the mouse and human putative promoters were identified. The presence of PU.1, SP1, MZF1, NF-κB, AML1 and OCT binding sites in the *Ptprj* promoter could explain the macrophage-specific expression of *Ptprj*. Many myeloid promoters have a functional PU.1 binding site upstream of the transcription start site. PU.1 binds to and recruits TATA binding protein (TBP), the primary component of the basal transcription factor TFIID in promoters lacking a TATA box [55], [72]. The expression of PU.1 has been shown to be important for macrophage differentiation and the expression of a number of molecules that mediate some of the immunological actions of macrophages [73]. Amongst other transcription factors that are involved in myeloid gene regulation and can interact with TBP are SP1 and OCT [55]. MZF1 expression is restricted to myeloid cell lines and is implicated in the development of cells of the myeloid lineage. The presence of a conserved MZF1 binding site in the *Ptprj* promoter is consistent with its expression in myeloid cells. The RNA binding zinc finger proteins EWS and FUS/TLS bind to the consensus binding sites for MZF1, implicating their significance in the assembly of the basal transcriptional complex [74]. Taken together, the bioinformatic analyses of the *Ptprj* putative promoter revealed that the *Ptprj* gene is highly regulated in macrophages and the *Ptprj* promoter resembles a macrophage-specific promoter.

IFNγ augments the immune response to LPS and other TLR agonists, thereby orchestrating distinct cellular signalling pathways through transcriptional control over a large number of genes [50]. In contrast, IFNγ priming of BMMs repressed *Ptprj* induction in response to LPS at early time points and had no effect at later time points (Figure 2E). Thus, *Ptprj* differs from many other macrophage-specific genes, which are regulated by both LPS and IFNγ. Many LPS-inducible genes including *Mpeg-1*, *Itm2b* and *Ramp2*, are repressed by CSF-1, but LPS-inducible in the presence of CSF-1 [75]. However, LPS-induction of *Ptprj* expression was more pronounced in the absence of CSF-1. In this respect, *Ptprj* regulation is similar to that of *Tlr9*, as absence of CSF-1 causes an elevation in *Tlr9* mRNA expression, but differs in response to IFNγ. In both mouse macrophages and human monocytes or dendritic cells, LPS induced CD148 expression whereas it repressed *c-fms* expression. Thus the increases in *Ptprj* gene expression are unlikely to be dependent on CSF-1 signalling, as LPS treatment not only suppresses expression of the CSF-1 receptor (C-fms) but also induces cleavage of the surface receptor making cells refractive to CSF-1 [75]. Dendritic cells are a heterogeneous population of cells with a number of different lineage relationships. *C-fms* is downregulated upon differentiation of monocytes into myeloid-derived dendritic cells and is also expressed at lower levels in other debdritic cell polulations [76]. Whilst expression is low in these cells, clearly, c-fms is functional since absence of CSF-1 results in >50% reduction in dendritic cell numbers [76], and CSF-1 can also regulate the number of tissue and blood elicited dendritic cells [77]–[79]. Thus ligation of CSF-1 to its receptor c-fms, provides both survival and differentiation signals for dendritic cells [80].

CD148 expression is up-regulated in chronic inflammatory diseases, such as Crohn’s disease and Cogan’s syndrome [61], [81]. The possible function of CD148 in inflammation could be similar to that of CD45, which plays an important role in lymphocyte development and function and has a crucial role in inflammation and cancer [82]. CD45 enhances T-cell and BCR signalling by dephosphorylating the auto-inhibitory phospho-tyrosine residues on Src family tyrosine kinases (SFKs) [83]. Indeed, recent studies of double knockout mice have revealed that CD45 and CD148 have overlapping functions, and can activate *src* family kinases in monocytes [84]; therefore activation or enhancement of CD148 expression could lead to modulation of the inflammatory response. The current results indicate that *Ptprj* is highly regulated by inflammatory stimuli *in vitro* and *in vivo*, however, the precise mechanism involved in the regulation of inflammation by *Ptprj* remains to be elucidated.
